# 1-(1-Benzofuran-2-yl)ethanone *O*-(4-chloro­benz­yl)oxime

**DOI:** 10.1107/S1600536812042675

**Published:** 2012-10-20

**Authors:** Tomasz Kosmalski, Andrzej K. Gzella

**Affiliations:** a Department of Organic Chemistry, Ludwik Rydygier Collegium Medicum in Bydgoszcz, Nicolaus Copernicus University in Torun, ul. A. Jurasza 2, 85-089 Bydgoszcz, Poland; b Department of Organic Chemistry, Poznan University of Medical Sciences, ul. Grunwaldzka 6, 60-780 Poznań, Poland; c, Faculty of Pharmacy, Ludwik Rydygier Collegium Medicum in Bydgoszcz, Nicolaus Copernicus University in Torun, ul. A. Jurasza 2, 85-089 Bydgoszcz, Poland

## Abstract

In the title compound, C_17_H_14_ClNO_2_, the *p*-chloro­benz­yloxy residue assumes an *E* conformation with respect to the benzofuran system. The carbo- and heterocyclic systems make a dihedral angle of 47.99 (4)°. In the crystal, there are no significant intermolecular interactions present.

## Related literature

For the biological activity of free oximes and their ethers, see: Chern *et al.* (2004 ▶); Emami *et al.* (2004 ▶); Demirayak *et al.* (2002 ▶); Bhandari *et al.* (2009 ▶); Jindal *et al.* (2003 ▶); Karakurt *et al.* (2001 ▶).
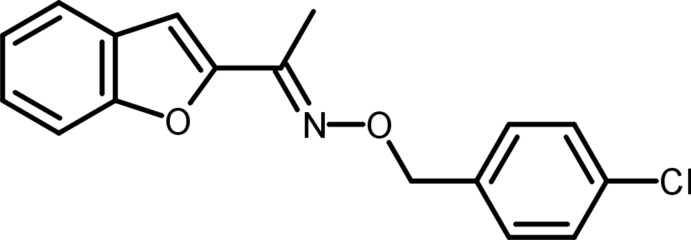


## Experimental

### 

#### Crystal data

C_17_H_14_ClNO_2_
*M* *_r_* = 299.74
Triclinic, 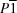

*a* = 5.8842 (4) Å
*b* = 7.1173 (6) Å
*c* = 17.2313 (16) Å
α = 93.802 (7)°
β = 97.998 (7)°
γ = 95.363 (7)°
*V* = 709.19 (10) Å^3^
*Z* = 2
Mo *K*α radiation
μ = 0.27 mm^−1^
*T* = 130 K
0.50 × 0.40 × 0.05 mm

#### Data collection

Agilent Xcalibur Atlas diffractometer
Absorption correction: multi-scan (*CrysAlis PRO*; Agilent, 2010 ▶) *T*_min_ = 0.783, *T*_max_ = 1.000
9366 measured reflections
3407 independent reflections
2817 reflections with *I* > 2σ(*I*)
*R*_int_ = 0.034

#### Refinement

*R*[*F* ^2^ > 2σ(*F* ^2^)] = 0.056
*wR*(*F* ^2^) = 0.137
*S* = 1.10
3407 reflections
191 parameters
H-atom parameters constrained
Δρ_max_ = 0.77 e Å^−3^
Δρ_min_ = −0.31 e Å^−3^

### 

Data collection: *CrysAlis PRO* (Agilent, 2010 ▶); cell refinement: *CrysAlis PRO*; data reduction: *CrysAlis PRO*; program(s) used to solve structure: *SHELXS97* (Sheldrick, 2008 ▶); program(s) used to refine structure: *SHELXL97* (Sheldrick, 2008 ▶); molecular graphics: *ORTEP-3 for Windows* (Farrugia, 1997 ▶); software used to prepare material for publication: *WinGX* (Farrugia, 1999 ▶), *PLATON* (Spek, 2009 ▶).

## Supplementary Material

Crystal structure: contains datablock(s) I, publication_text. DOI: 10.1107/S1600536812042675/bt6848sup1.cif

Structure factors: contains datablock(s) I. DOI: 10.1107/S1600536812042675/bt6848Isup2.hkl

Supplementary material file. DOI: 10.1107/S1600536812042675/bt6848Isup3.cml

Additional supplementary materials:  crystallographic information; 3D view; checkCIF report

## Figures and Tables

**Fig. 1.**
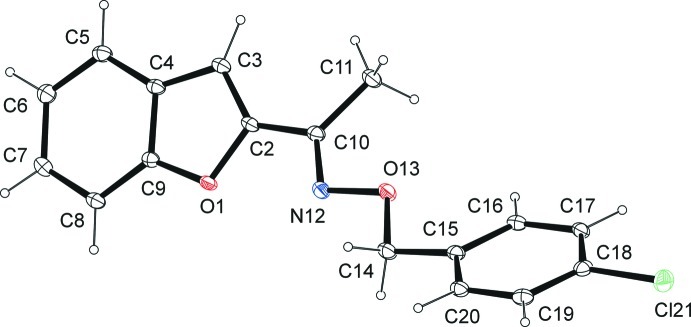
The molecular structure of the title compound showing the atomic labelling scheme. Non-H atoms are drawn as 30% probability displacement ellipsoids; H atoms are shown as small spheres of arbitrary radius.

**Fig. 2.**
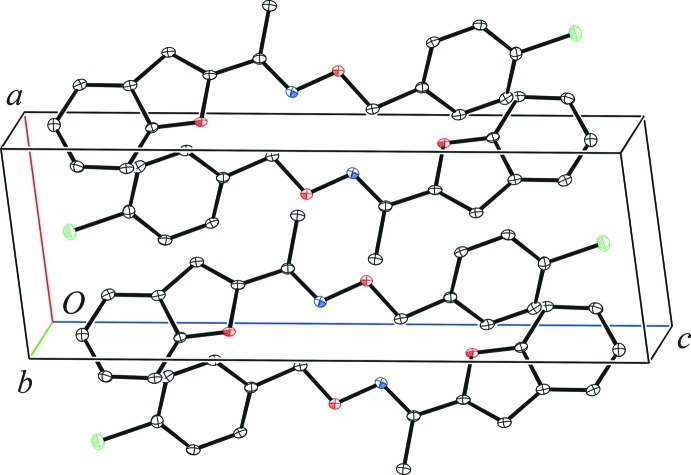
The crystal packing of the title compound. H atoms have been omitted for clarity.

## supplementary crystallographic information

### Comment

The study of free oximes and their ethers have become of much interest in recent years on account of their diverse biological activities. Antienteroviral, antifungal, antibacterial, antineoplastic, anticonvulsant and antimicrobial activities (Chern *et al.*, 2004; Emami *et al.*, 2004; Demirayak *et al.*, 2002; Bhandari *et al.*, 2009; Jindal *et al.*, 2003; Karakurt *et al.*, 2001) are a few among many other activities.

The crystal structure investigation of the title compound was undertaken in order to obtain information about the spatial structure of the molecule.

The molecular structure of the title compound and the atom-labelling scheme is illustrated in Fig. 1.

The nine-membered benzofuran system is almost planar with an r.m.s. deviation of 0.0122 Å. The *p*-chlorobenzyloxy moiety is in *E* configuration with respect to the C2 atom of the benzofuran system. This arrangement is confirmed by the torsional angle C2—C10—N12—O13 of 179.91 (17)°. Simultaneously, the torsion angle C10—N12—O13—C14, 173.34 (18)°, reveals an antiperiplanar conformation for atoms C10 and C14. Furthermore, the dihedral angle made by the mean planes of the above mentioned systems amounts to 47.99 (4)°.

The main factor that determines the crystal packing are normal van der Waals interactions.

### Refinement

All H atoms were set to idealized positions and were refined with the riding model approximation: C_methyl_—H = 0.96 Å, C_methylene_—H = 0.97 Å, C(*sp*^2^)—H = 0.93 Å; *U*_iso_(H) = 1.2*U*_eq_(C) or 1.5*U*_eq_(C) for methyl H. The methyl group was refined as rigid group which was allowed to rotate.

### Crystal data

**Table:**

C_17_H_14_ClNO_2_	*Z* = 2
*M**_r_* = 299.74	*F*(000) = 312
Triclinic, *P*1	*D*_x_ = 1.404 Mg m^−^^3^
Hall symbol: -P 1	Melting point = 377–378 K
*a* = 5.8842 (4) Å	Mo *K*α radiation, λ = 0.71073 Å
*b* = 7.1173 (6) Å	Cell parameters from 3128 reflections
*c* = 17.2313 (16) Å	θ = 2.4–28.9°
α = 93.802 (7)°	µ = 0.27 mm^−^^1^
β = 97.998 (7)°	*T* = 130 K
γ = 95.363 (7)°	Lath, colourless
*V* = 709.19 (10) Å^3^	0.50 × 0.40 × 0.05 mm

### Data collection

**Table:**

Agilent Xcalibur Atlas diffractometer	3407 independent reflections
Radiation source: Enhance (Mo) X-ray Source	2817 reflections with *I* > 2σ(*I*)
Graphite monochromator	*R*_int_ = 0.034
ω scans	θ_max_ = 29.0°, θ_min_ = 2.4°
Absorption correction: multi-scan (*CrysAlis PRO*; Agilent, 2010)	*h* = −8→7
*T*_min_ = 0.783, *T*_max_ = 1.000	*k* = −9→9
9366 measured reflections	*l* = −22→23

### Refinement

**Table:**

Refinement on *F*^2^	Primary atom site location: structure-invariant direct methods
Least-squares matrix: full	Secondary atom site location: difference Fourier map
*R*[*F*^2^ > 2σ(*F*^2^)] = 0.056	Hydrogen site location: inferred from neighbouring sites
*wR*(*F*^2^) = 0.137	H-atom parameters constrained
*S* = 1.10	*w* = 1/[σ^2^(*F*_o_^2^) + (0.0487*P*)^2^ + 0.7613*P*] where *P* = (*F*_o_^2^ + 2*F*_c_^2^)/3
3407 reflections	(Δ/σ)_max_ < 0.001
191 parameters	Δρ_max_ = 0.77 e Å^−^^3^
0 restraints	Δρ_min_ = −0.31 e Å^−^^3^

### Special details

**Table:**

Geometry. All e.s.d.'s (except the e.s.d. in the dihedral angle between two l.s. planes) are estimated using the full covariance matrix. The cell e.s.d.'s are taken into account individually in the estimation of e.s.d.'s in distances, angles and torsion angles; correlations between e.s.d.'s in cell parameters are only used when they are defined by crystal symmetry. An approximate (isotropic) treatment of cell e.s.d.'s is used for estimating e.s.d.'s involving l.s. planes.
Refinement. Refinement of *F*^2^ against ALL reflections. The weighted *R*-factor *wR* and goodness of fit *S* are based on *F*^2^, conventional *R*-factors *R* are based on *F*, with *F* set to zero for negative *F*^2^. The threshold expression of *F*^2^ > σ(*F*^2^) is used only for calculating *R*-factors(gt) *etc*. and is not relevant to the choice of reflections for refinement. *R*-factors based on *F*^2^ are statistically about twice as large as those based on *F*, and *R*- factors based on ALL data will be even larger.

### Fractional atomic coordinates and isotropic or equivalent isotropic displacement parameters (Å^2^)

**Table:**

	*x*	*y*	*z*	*U*_iso_*/*U*_eq_	
O1	0.8938 (2)	0.1648 (2)	0.67864 (8)	0.0185 (3)	
C2	0.6830 (4)	0.2349 (3)	0.65738 (13)	0.0178 (4)	
C3	0.5839 (4)	0.2838 (3)	0.72112 (13)	0.0190 (4)	
H3	0.4404	0.3350	0.7210	0.023*	
C4	0.7371 (4)	0.2436 (3)	0.78881 (13)	0.0188 (4)	
C5	0.7372 (4)	0.2580 (3)	0.87016 (13)	0.0218 (5)	
H5	0.6144	0.3090	0.8921	0.026*	
C6	0.9194 (4)	0.1967 (3)	0.91800 (13)	0.0242 (5)	
H6	0.9209	0.2054	0.9733	0.029*	
C7	1.1032 (4)	0.1214 (3)	0.88624 (13)	0.0228 (5)	
H7	1.2259	0.0798	0.9205	0.027*	
C8	1.1080 (4)	0.1070 (3)	0.80611 (13)	0.0200 (4)	
H8	1.2312	0.0567	0.7843	0.024*	
C9	0.9240 (4)	0.1697 (3)	0.75924 (12)	0.0171 (4)	
C10	0.6054 (4)	0.2459 (3)	0.57402 (13)	0.0190 (4)	
C11	0.3554 (4)	0.2650 (4)	0.54716 (14)	0.0249 (5)	
H11A	0.3417	0.3809	0.5200	0.037*	
H11B	0.2732	0.2712	0.5928	0.037*	
H11C	0.2881	0.1553	0.5112	0.037*	
N12	0.7611 (3)	0.2386 (3)	0.52841 (10)	0.0206 (4)	
O13	0.6618 (3)	0.2504 (2)	0.44972 (9)	0.0224 (4)	
C14	0.8445 (4)	0.2641 (3)	0.40298 (13)	0.0230 (5)	
H14A	0.9267	0.1491	0.4058	0.028*	
H14B	0.9564	0.3751	0.4231	0.028*	
C15	0.7438 (4)	0.2847 (3)	0.31948 (12)	0.0188 (4)	
C16	0.5159 (4)	0.2194 (3)	0.28902 (13)	0.0194 (4)	
H16	0.4154	0.1687	0.3228	0.023*	
C17	0.4342 (4)	0.2276 (3)	0.20982 (13)	0.0196 (4)	
H17	0.2790	0.1825	0.1893	0.024*	
C18	0.5821 (4)	0.3024 (3)	0.16110 (12)	0.0205 (4)	
C19	0.8082 (4)	0.3733 (3)	0.19012 (13)	0.0214 (5)	
H19	0.9070	0.4265	0.1564	0.026*	
C20	0.8865 (4)	0.3647 (3)	0.26924 (13)	0.0202 (4)	
H20	1.0403	0.4141	0.2898	0.024*	
Cl21	0.48371 (11)	0.30347 (9)	0.06126 (3)	0.03010 (18)	

### Atomic displacement parameters (Å^2^)

**Table:**

	*U*^11^	*U*^22^	*U*^33^	*U*^12^	*U*^13^	*U*^23^
O1	0.0128 (7)	0.0216 (8)	0.0222 (7)	0.0061 (6)	0.0031 (6)	0.0021 (6)
C2	0.0121 (10)	0.0152 (10)	0.0266 (11)	0.0033 (8)	0.0025 (8)	0.0025 (8)
C3	0.0134 (10)	0.0183 (10)	0.0261 (11)	0.0031 (8)	0.0039 (8)	0.0028 (8)
C4	0.0146 (10)	0.0155 (10)	0.0266 (11)	0.0015 (8)	0.0048 (8)	0.0007 (8)
C5	0.0174 (11)	0.0226 (11)	0.0263 (11)	0.0029 (9)	0.0067 (9)	0.0006 (9)
C6	0.0239 (12)	0.0281 (12)	0.0210 (11)	0.0040 (9)	0.0033 (9)	0.0018 (9)
C7	0.0176 (11)	0.0241 (12)	0.0266 (11)	0.0037 (9)	0.0006 (9)	0.0052 (9)
C8	0.0145 (10)	0.0176 (11)	0.0283 (11)	0.0029 (8)	0.0038 (9)	0.0020 (8)
C9	0.0139 (10)	0.0148 (10)	0.0226 (10)	−0.0001 (8)	0.0045 (8)	0.0011 (8)
C10	0.0164 (10)	0.0164 (10)	0.0244 (11)	0.0037 (8)	0.0030 (8)	0.0010 (8)
C11	0.0169 (11)	0.0315 (13)	0.0267 (11)	0.0068 (9)	0.0015 (9)	0.0032 (9)
N12	0.0173 (9)	0.0251 (10)	0.0198 (9)	0.0050 (7)	0.0011 (7)	0.0025 (7)
O13	0.0158 (8)	0.0330 (9)	0.0192 (8)	0.0062 (6)	0.0025 (6)	0.0037 (6)
C14	0.0147 (10)	0.0314 (13)	0.0239 (11)	0.0054 (9)	0.0042 (9)	0.0021 (9)
C15	0.0168 (10)	0.0170 (11)	0.0233 (11)	0.0047 (8)	0.0036 (8)	0.0006 (8)
C16	0.0159 (10)	0.0187 (11)	0.0251 (11)	0.0037 (8)	0.0068 (8)	0.0022 (8)
C17	0.0145 (10)	0.0181 (10)	0.0259 (11)	0.0040 (8)	0.0013 (8)	−0.0003 (8)
C18	0.0248 (11)	0.0174 (11)	0.0202 (10)	0.0074 (9)	0.0041 (9)	0.0005 (8)
C19	0.0206 (11)	0.0182 (11)	0.0273 (11)	0.0027 (8)	0.0087 (9)	0.0030 (8)
C20	0.0145 (10)	0.0179 (11)	0.0278 (11)	0.0009 (8)	0.0044 (8)	−0.0014 (8)
Cl21	0.0352 (4)	0.0342 (3)	0.0212 (3)	0.0077 (3)	0.0019 (2)	0.0033 (2)

### Geometric parameters (Å, º)

**Table:**

O1—C9	1.373 (2)	C11—H11B	0.9800
O1—C2	1.391 (2)	C11—H11C	0.9800
C2—C3	1.354 (3)	N12—O13	1.411 (2)
C2—C10	1.455 (3)	O13—C14	1.430 (3)
C3—C4	1.433 (3)	C14—C15	1.500 (3)
C3—H3	0.9500	C14—H14A	0.9900
C4—C5	1.399 (3)	C14—H14B	0.9900
C4—C9	1.405 (3)	C15—C16	1.395 (3)
C5—C6	1.382 (3)	C15—C20	1.399 (3)
C5—H5	0.9500	C16—C17	1.389 (3)
C6—C7	1.411 (3)	C16—H16	0.9500
C6—H6	0.9500	C17—C18	1.386 (3)
C7—C8	1.382 (3)	C17—H17	0.9500
C7—H7	0.9500	C18—C19	1.389 (3)
C8—C9	1.385 (3)	C18—Cl21	1.738 (2)
C8—H8	0.9500	C19—C20	1.384 (3)
C10—N12	1.289 (3)	C19—H19	0.9500
C10—C11	1.501 (3)	C20—H20	0.9500
C11—H11A	0.9800		
			
C9—O1—C2	105.57 (15)	H11A—C11—H11B	109.5
C3—C2—O1	111.63 (18)	C10—C11—H11C	109.5
C3—C2—C10	130.7 (2)	H11A—C11—H11C	109.5
O1—C2—C10	117.67 (17)	H11B—C11—H11C	109.5
C2—C3—C4	106.82 (19)	C10—N12—O13	110.10 (17)
C2—C3—H3	126.6	N12—O13—C14	107.89 (15)
C4—C3—H3	126.6	O13—C14—C15	108.84 (17)
C5—C4—C9	118.6 (2)	O13—C14—H14A	109.9
C5—C4—C3	135.9 (2)	C15—C14—H14A	109.9
C9—C4—C3	105.50 (19)	O13—C14—H14B	109.9
C6—C5—C4	118.7 (2)	C15—C14—H14B	109.9
C6—C5—H5	120.7	H14A—C14—H14B	108.3
C4—C5—H5	120.7	C16—C15—C20	118.7 (2)
C5—C6—C7	121.3 (2)	C16—C15—C14	122.46 (19)
C5—C6—H6	119.4	C20—C15—C14	118.80 (19)
C7—C6—H6	119.4	C17—C16—C15	120.7 (2)
C8—C7—C6	121.2 (2)	C17—C16—H16	119.6
C8—C7—H7	119.4	C15—C16—H16	119.6
C6—C7—H7	119.4	C18—C17—C16	119.2 (2)
C7—C8—C9	116.6 (2)	C18—C17—H17	120.4
C7—C8—H8	121.7	C16—C17—H17	120.4
C9—C8—H8	121.7	C17—C18—C19	121.4 (2)
O1—C9—C8	125.81 (19)	C17—C18—Cl21	119.18 (17)
O1—C9—C4	110.48 (18)	C19—C18—Cl21	119.38 (17)
C8—C9—C4	123.7 (2)	C20—C19—C18	118.7 (2)
N12—C10—C2	116.27 (19)	C20—C19—H19	120.7
N12—C10—C11	124.8 (2)	C18—C19—H19	120.7
C2—C10—C11	118.90 (18)	C19—C20—C15	121.3 (2)
C10—C11—H11A	109.5	C19—C20—H20	119.3
C10—C11—H11B	109.5	C15—C20—H20	119.3
			
C9—O1—C2—C3	0.5 (2)	O1—C2—C10—N12	−17.9 (3)
C9—O1—C2—C10	−179.91 (18)	C3—C2—C10—C11	−18.3 (4)
O1—C2—C3—C4	−0.2 (2)	O1—C2—C10—C11	162.22 (19)
C10—C2—C3—C4	−179.7 (2)	C2—C10—N12—O13	179.91 (17)
C2—C3—C4—C5	−179.0 (2)	C11—C10—N12—O13	−0.3 (3)
C2—C3—C4—C9	−0.2 (2)	C10—N12—O13—C14	173.34 (18)
C9—C4—C5—C6	−0.9 (3)	N12—O13—C14—C15	−177.74 (17)
C3—C4—C5—C6	177.7 (2)	O13—C14—C15—C16	−24.9 (3)
C4—C5—C6—C7	0.2 (3)	O13—C14—C15—C20	158.05 (19)
C5—C6—C7—C8	0.3 (4)	C20—C15—C16—C17	2.1 (3)
C6—C7—C8—C9	−0.1 (3)	C14—C15—C16—C17	−174.9 (2)
C2—O1—C9—C8	177.8 (2)	C15—C16—C17—C18	−0.2 (3)
C2—O1—C9—C4	−0.7 (2)	C16—C17—C18—C19	−1.5 (3)
C7—C8—C9—O1	−178.81 (19)	C16—C17—C18—Cl21	177.27 (16)
C7—C8—C9—C4	−0.6 (3)	C17—C18—C19—C20	1.3 (3)
C5—C4—C9—O1	179.55 (18)	Cl21—C18—C19—C20	−177.53 (16)
C3—C4—C9—O1	0.6 (2)	C18—C19—C20—C15	0.7 (3)
C5—C4—C9—C8	1.1 (3)	C16—C15—C20—C19	−2.4 (3)
C3—C4—C9—C8	−177.9 (2)	C14—C15—C20—C19	174.7 (2)
C3—C2—C10—N12	161.5 (2)		
